# The Combination of APRI and ALBI Facilitates Preoperative Risk Stratification for Patients Undergoing Liver Surgery After Neoadjuvant Chemotherapy

**DOI:** 10.1245/s10434-018-07125-6

**Published:** 2019-01-07

**Authors:** D. Pereyra, B. Rumpf, M. Ammann, S. F. Perrodin, D. Tamandl, C. Haselmann, J. Stift, C. Brostjan, F. Laengle, G. Beldi, T. Gruenberger, P. Starlinger

**Affiliations:** 1 Department of Surgery, General Hospital, Medical University of Vienna, Vienna, Austria; 2 Department of Surgery, State Hospital Wiener Neustadt, Wiener Neustadt, Austria; 3 Department of Visceral Surgery and Medicine, University of Bern, Inselspital, Bern, Switzerland; 4 Department of Biomedical Imaging and Image-Guided Therapy, General Hospital, Medical University of Vienna, Vienna, Austria; 5 Clinical Institute of Pathology, General Hospital, Medical University of Vienna, Vienna, Austria; 6 Department of Surgery, Rudolfstiftung Hospital, Vienna, Austria; 7 Present Address: Department of Surgery, Kaiser Franz Josef Hospital, Vienna, Austria

## Abstract

**Background:**

Neoadjuvant chemotherapy (NeoCTx) is performed for most patients with colorectal cancer liver metastases (CRCLM). However, chemotherapy-associated liver injury (CALI) has been associated with poor postoperative outcome. To date, however, no clinically applicable and noninvasive tool exists to assess CALI before liver resection.

**Methods:**

Routine blood parameters were assessed in 339 patients before and after completion of NeoCTx and before surgery. The study assessed the prognostic potential of the aspartate aminotransferase (AST)-to-platelet ratio index (APRI), the albumin-bilirubin grade (ALBI), and their combinations. Furthermore, an independent multi-center validation cohort (*n* = 161) was included to confirm the findings concerning the prediction of postoperative outcome.

**Results:**

Higher ALBI, APRI, and APRI + ALBI were found in patients with postoperative morbidity (*P* = 0.001, *P* = 0.064, *P* = 0.001, respectively), liver dysfunction (LD) (*P* = 0.009, *P* = 0.012, *P* < 0.001), or mortality (*P* = 0.037, *P* = 0.045, *P* = 0.016), and APRI + ALBI had the highest predictive potential for LD (area under the curve [AUC], 0.695). An increase in APRI + ALBI was observed during NeoCTx (*P* < 0.001). Patients with longer periods between NeoCTx and surgery showed a greater decrease in APRI + ALBI (*P* = 0.006) and a trend for decreased CALI at surgery. A cutoff for APRI + ALBI at − 2.46 before surgery was found to identify patients with CALI (*P* = 0.002) and patients at risk for a prolonged hospital stay (*P* = 0.001), intensive care (*P* < 0.001), morbidity (*P* < 0.001), LD (*P* < 0.001), and mortality (*P* = 0.021). Importantly, the study was able to confirm the predictive potential of APRI + ALBI for postoperative LD and mortality in a multicenter validation cohort.

**Conclusion:**

Determination of APRI + ALBI before surgery enables identification of high-risk patients for liver resection. The combined score seems to dynamically reflect CALI. Thus, APRI + ALBI could be a clinically relevant tool for optimizing timing of surgery in CRCLM patients after NeoCTx.

**Electronic supplementary material:**

The online version of this article (10.1245/s10434-018-07125-6) contains supplementary material, which is available to authorized users.

Throughout the past decade, multimodal management of patients with liver metastases of colorectal carcinoma (CRCLM) has improved, leading to a significant advancement in oncologic outcome.^1^^,^^2^ Liver resection (LR) is considered the only potentially curative treatment of colorectal liver metastases if complete resection can be achieved. In this context, neoadjuvant chemotherapy (NeoCTx) has proved to be efficient as a method to downsize liver metastases, which makes curative LR feasible for a greater number of patients.^3^–^5^ Moreover, in addition, findings have shown that patients with primary resectable CRCLM benefit from NeoCTx.^6^–^8^ However, preoperative treatment with oxaliplatin or irinotecan, the most commonly used chemotherapeutic agents for patients with CRCLM, is known to cause chemotherapy-associated liver injury (CALI),^9^ ranging from steatosis to more severe liver damage such as sinusoidal obstruction syndrome (SOS) and chemotherapy-associated steatohepatitis (CASH).^10^ Importantly, the development of postoperative liver dysfunction (LD) has been associated with a higher incidence of morbidity and mortality as well as the presence of CASH and SOS.^11^–^15^ Thus, the amount of NeoCTx has to be optimally balanced between best oncologic efficacy and least induced liver damage.^16^^,^^17^ Unfortunately, assessment and staging of CALI remains challenging, and a clinically applicable tool for noninvasive evaluation still is missing.

Recently, a number of noninvasive scoring systems for staging chronic liver disease have been established based on routine laboratory parameters. Among these, the aspartate-to-platelet-ratio index (APRI) and the albumin-bilirubin grade (ALBI) have been shown to allow estimation of liver function, as reflected by the Child–Pugh score and indocyanine green (ICG) clearance.^18^–^25^

Although APRI was first introduced as a noninvasive marker for hepatitis C-related liver fibrosis,^26^^,^^27^ it evolved as a general marker for liver function in fibrotic and cirrhotic patients.^28^ In addition, findings have shown APRI to be associated with SOS in patients after oxaliplatin-based chemotherapy.^29^^,^^30^ Similarly, ALBI has been established as a grading system for hepatic function, especially in patients with hepatocellular carcinoma (HCC).^31^^,^^32^

Interestingly, studies have found both APRI and ALBI to be predictors of postoperative outcome for patients undergoing liver surgery.^33^–^36^ In particular, studies have shown APRI to be predictive for the development of postoperative LD in patients after major LR, and an association with the presence of SOS has been suggested.^34^^,^^35^ In parallel, studies have shown ALBI to be a predictive marker for postoperative liver failure in patients undergoing LR for HCC.^37^^,^^38^

This study aimed to assess the potential of APRI and ALBI to predict a poor postoperative outcome for a homogeneous cohort of CRCLM patients undergoing LR. In addition, the study aimed to assess the potential predictive benefit of APRI and ALBI combined and to elucidate the relation of both markers to NeoCTx.

## Methods

### Patients

The study enrolled patients undergoing LR between 2001 and 2014 at the Medical University of Vienna who had CRCLM. Data including routine blood parameters were prospectively collected. Information on NeoCTx was documented, and if applicable, blood parameters were assessed before chemotherapy (preNeoCTx) and after completion of chemotherapy (postNeoCTx). Blood was taken from all patients before LR (preOP). Both APRI and ALBI were calculated as specified in Fig. S1.

The current study aimed to assess CALI by a rigorous pathologic examination of 170 patients. In addition, the study included a validation cohort recruited at four different institutions (Medical University of Vienna, Rudolfstiftung Hospital Vienna, State Hospital Wiener Neustadt, Inselspital, and University Hospital Bern). Based on data from the exploration cohort, a sample size calculation was performed, and 158 patients were found to be suitable for validation, based on the proportion of patients with LD in the defined risk groups (*α* = 5%, *β* = 80%).

All the patients gave written informed consent. The study was conducted in accordance with the Declaration of Helsinki and approved by the institutional ethics committee (#424/2010; #2032/2013).

### Statistical Analysis

Statistical analyses, based on nonparametric tests, were performed using SPSS (version 23; IBM Corp, Armonk, NY, USA). A *P* value lower than 0.05 was considered statistically significant. For more information on statistical methods refer to the supplementary material.

## Results

### Patient Demographics

Data from 339 patients with CRCLM were collected. The data were prospectively recorded in the institutional database. Patients were included independently of NeoCTx regimen and grouped as “no NeoCTx,” “low hepatotoxicity” including fluorouracil-based regimen, “oxaliplatin,” “irinotecan,” and “oxaliplatin + irinotecan.” Baseline characteristics are presented in Table S1.

### ALBI, APRI and APRI + ALBI are Elevated in Patients With Postoperative Morbidity, LD, or Mortality

**Fig. 1:**
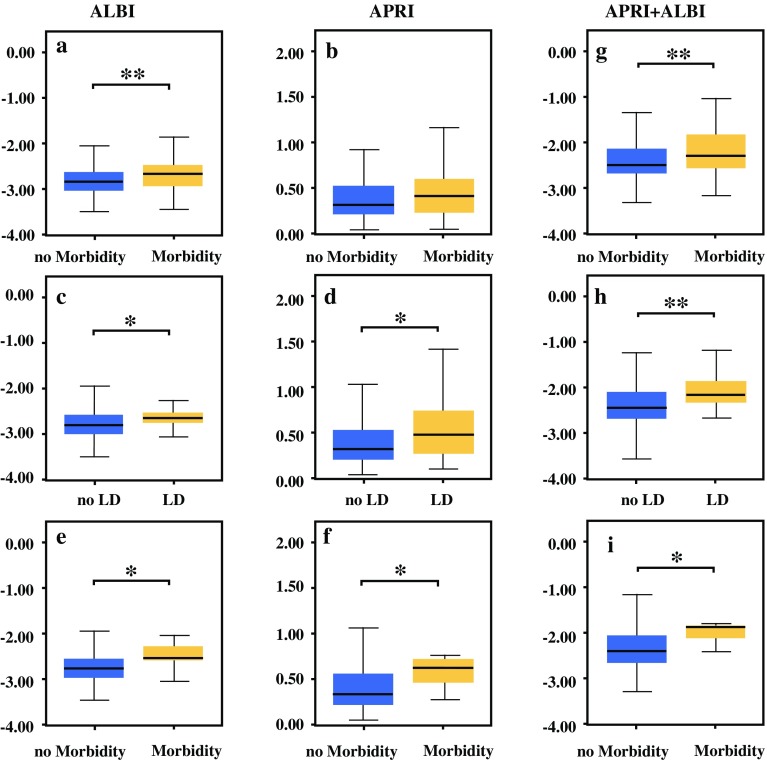
Albumin-bilirubin grade (ALBI), aspartate aminotransferase (AST)-to-platelet ratio index (APRI), and APRI + ALBI in accordance with clinical outcome after liver resection. Levels of ALBI (**a, c, e**), APRI (**b, d, f**), and APRI + ALBI (**g, h, i**) are shown for patients with and without postoperative morbidity (**a, b, g**), for patients with and without postoperative liver dysfunction (LD) (**c, d, h**), and for patients with and those without postoperative mortality (**e, f, i**). *P < 0.05. **P < 0.005

First, the study aimed to validate previous reports on the predictive potential of preoperative APRI and ALBI concerning postoperative morbidity, LD, and mortality. Accordingly, patients with postoperative morbidity showed higher preoperative levels of ALBI (median no morbidity, − 2.84; median morbidity, − 2.68; *P* = 0.001; Fig. 1a) and a tendency toward higher levels of APRI (median no morbidity, 0.33; median morbidity, 0.43; *P* = 0.064; Fig. 1b). Similarly, patients who experienced postoperative LD had higher values for ALBI (median no LD, − 2.82; median LD, − 2.66; *P* = 0.009; Fig. 1c) and APRI (median no LD, 0.33; median LD, 0.49; *P* = 0.012; Fig. 1d) before LR. Notably, seven patients died within 90 postoperative days. Compared with the patients who did not die postoperatively, the patients who died displayed higher preoperative levels of ALBI (median no mortality, − 2.79; median mortality, − 2.55; *P* = 0.037; Fig. 1e) and APRI (median no mortality, 0.33; median mortality, 0.62; *P* = 0.045; Fig. 1f).

Subsequently, we aimed to compare mathematical combinations of APRI and ALBI and to assess the differences between patients with and without morbidity, LD, or mortality. According to our hypothesis, a summative combination of APRI and ALBI should be the most effective variant because this combination should theoretically increase the discriminatory capacity of each individual marker. Indeed, the patients with postoperative morbidity showed higher values of APRI + ALBI already before the operation (median no morbidity, − 2.48; median morbidity, − 2.27; *P* = 0.001; Fig. 1g). This also was found for patients who would experience LD (median no LD, − 2.44; median LD, − 2.16; *P* < 0.001; Fig. 1h) and for patients who did not survive for 90 postoperative days (median no mortality, − 2.41; median mortality, − 1.87; *P* = 0.016; Fig. 1i). Notably, other mathematical combinations were assessed but ultimately found to be of less or no statistical significance, as illustrated in Fig. S2.

### APRI and ALBI Combined Improves the Prognostic Potential for Prediction of Postoperative LD

To compare the potential of APRI and ALBI alone with that of both variables combined to discriminate between patients with and without LD, receiver operating characteristic (ROC) curve analysis was performed. Notably, comparable results were shown by ALBI (area under the curve [AUC], 0.636; 95% confidence interval [CI], 0.552–0.720; *P* = 0.009) and APRI alone (AUC, 0.633; 95% CI, 0.532–0.734; *P* = 0.012). Still, APRI and ALBI combined showed the highest discriminatory potential, with an AUC of 0.695 (95% CI, 0.613–0.777; *P* < 0.001). Thus, further analysis was focused on APRI + ALBI.

### A Cutoff of APRI + ALBI at − 2.46 Allows Preoperative Risk Stratification of Patients Undergoing Liver Resection

**Fig. 2:**
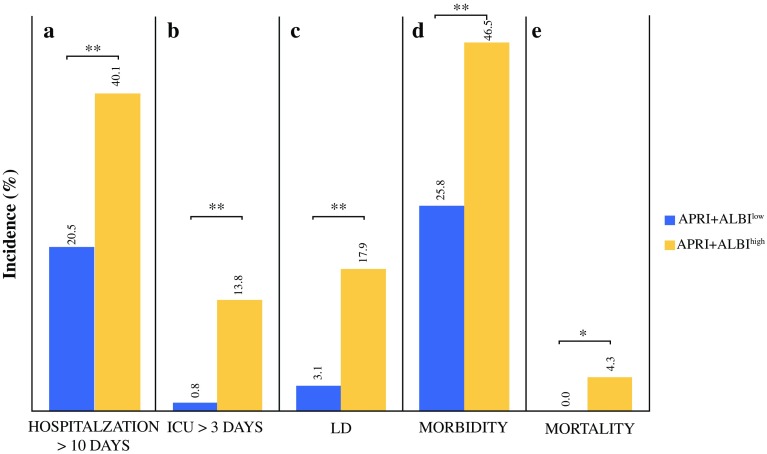
Frequencies of adverse clinical outcome for patients above and below the proposed cutoff of–2.46 for APRI + ALBI. Incidences of (**a**) prolonged hospitalization, (**b**) prolonged intensive care unit (ICU) stay, (**c**) liver dysfunction (LD), (**d**) morbidity, and (**e**) mortality are shown according to the proposed risk groups (APRI + ALBI^low/high^). *P < 0.05. **P < 0.005. APRI, aspartate aminotransferase (AST)-to-platelet ratio index; ALBI, albumin-bilirubin grade

Based on the ROC curve of APRI + ALBI for postoperative LD, a cutoff was identified using Youden’s J statistic. This cutoff was found to be − 2.46. Accordingly, the patients were divided into a low-risk group (APRI + ALBI^low^, ≤ 2.46) and a high-risk group (APRI + ALBI^high^, > 2.46). For the patients above the cutoff, a significantly higher incidences of prolonged hospitalization (> 10 days) (APRI + ALBI^high^ 40.1% vs APRI + ALBI^low^ 20.5%; *P* < 0.001; Fig. 2a), and prolonged intensive care unit (ICU) stay (> 3 days) (APRI + ALBI^high^ 13.8% vs APRI + ALBI^low^ 0.8%; *P* < 0.001; Fig. 2b) were observed. Furthermore, the patients in the high-risk group displayed significantly higher incidences of postoperative LD (APRI + ALBI^high^ 17.9% vs APRI + ALBI^low^ 3.1%; *P* < 0.001; Fig. 2c) and morbidity (APRI + ALBI^high^ 46.5% vs APRI + ALBI^low^ 25.8%; *P *< 0.001; Fig. 2d).

Interestingly, only the patients in the APRI + ALBI^high^ group experienced clinically relevant postoperative LD graded according to the International Study Group of Liver Surgery (ISGLS) criteria (grades B and C), as visualized in Fig. S3a. Similarly, severe postoperative complications classified higher than Dindo grade 4 were exclusively observed in high-risk patients according to APRI + ALBI (Fig. S3b). Ultimately, only patients in the high-risk group died within 90 postoperative days (*P* = 0.021; Fig. 2e). Notably, the study was able to confirm the prediction of a poor outcome for both minor and major LR (Fig. S4).

### Prediction of Postoperative LD Using APRI + ALBI is Independent of Other Variables and Confounders

To test for the independence of the proposed cutoff from other predictors of LD and to identify potential confounding factors, multivariable analysis was performed (Table S2). However, after the proposed cutoff for APRI + ALBI, the extent of resection, the alkaline phosphatase (AP) level, the gamma-glutamyl transferase (GGT) level, and the retention rate after 15 min for ICG clearance were entered into the multivariable model, only the proposed cutoff (*P* = 0.002; odds ratio [OR], 5.501; 95% CI, 1.822–16.606) and preoperative gGT (*P* < 0.001; OR, 1.006; 95% CI, 1.003–1.010) remained as significant independent variables after stepwise forward selection.

### Patients Treated with Neoadjuvant Chemotherapy Display Higher Levels of Preoperative APRI + ALBI

**Fig. 3:**
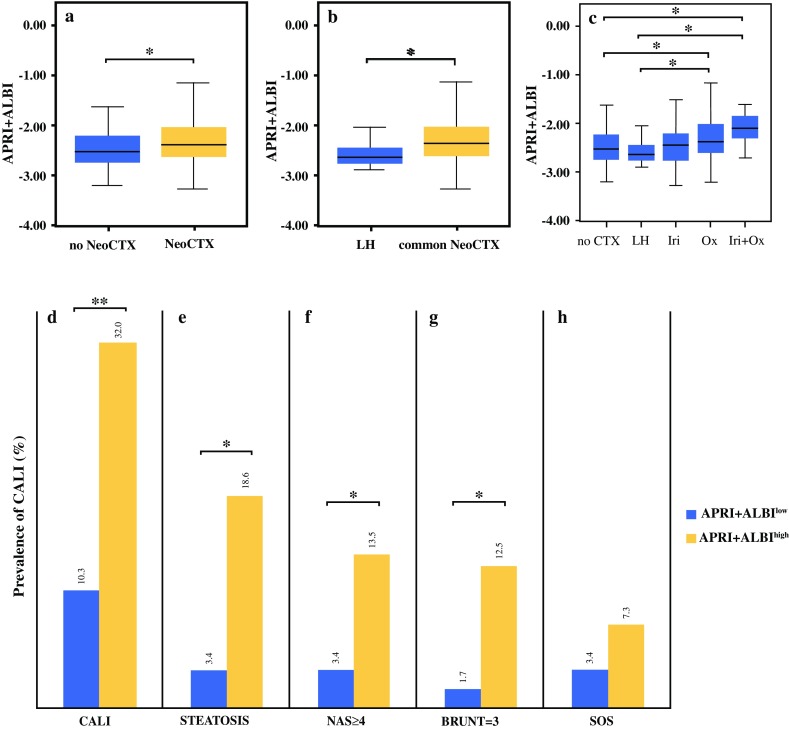
Patients above the proposed cutoff of – 2.46 for APRI + ALBI show a higher prevalence of chemotherapy-associated liver injury (CALI). Levels of APRI + ALBI are shown for patients who were or were not treated with neoadjuvant chemotherapy (NeoCTx). Patients with (**a**) less hepatotoxic chemotherapy (for detailed information refer to Table S1) compared with (**b**) more hepatotoxic (irinotecan/oxaliplatin-based) NeoCTx, and (**c**) according to the type of NeoCTx. Furthermore, prevalence of (**d**) CALI, (**e**) steatosis, (**f**) chemotherapy-associated steatohepatitis according to nonalcoholic fatty liver disease activity score (NAS) and Brunt et al.^40^, and (**g**) sinusoidal obstruction syndrome (SOS) are shown for patients with high or low values of preoperative APRI + ALBI. APRI, aspartate aminotransferase (AST)-to-platelet ratio index; ALBI, albumin-bilirubin grade; LH, less hepatotoxic chemotherapeutic regimens; Iri, irinotecan; Ox, oxaliplatin. *P < 0.05. **P < 0.005

Because APRI + ALBI were associated with postoperative outcome, we aimed to investigate the underlying etiology behind elevated levels of APRI + ALBI. Increased levels of APRI + ALBI were observed in patients treated with NeoCTx (median no NeoCTx, − 2.53; median NeoCTx, − 2.39; *P* = 0.028; Fig. 3a). Intriguingly, patients treated with chemotherapeutic agents with lower hepatotoxicity also showed significantly lower scores than the patients treated with commonly used regimens (i.e., based on oxaliplatin, irinotecan, or a combination of both) (median low hepatotoxicity, − 2.64; median hepatotoxic NeoCTx, − 2.37; *P* = 0.038; Fig. 3b). Notably, APRI + ALBI did not differ significantly between the patients without NeoCTx and the patients treated with fewer hepatotoxic agents (*P* = 0.641; Fig. 3c). Strikingly, a clear distinction was observed between the patients treated with different types of common regimens and the patients treated with less or no hepatotoxic NeoCTx (mean no NeoCTx, − 2.53; mean irinotecan, − 2.43 [*P* = 0.616]; median oxaliplatin, − 2.38 [*P* = 0.011]; median oxaliplatin + irinotecan, − 2.01 [*P* = 0.019]; median low hepatotoxicity, − 2.64 [*P* = 0.369, *P* = 0.024, *P* = 0.044, respectively]; Fig. 3c).

### Patients with CALI Can be Identified Using the Proposed Cutoff for APRI + ALBI

Next, the relation of the proposed cutoff for CALI was assessed. A significantly higher prevalence of CALI irrespective of type was observed for the patients in the high-risk group (APRI + ALBI^high^ 32% vs APRI + ALBI^low^ 10.3%; *P* = 0.002; Fig. 3d). The subtypes of CALI, patients in the high-risk group were found more frequently to display severe steatosis (APRI + ALBI^high^ 18.6% vs APRI + ALBI^low^ 3.4%; *P* = 0.007; Fig. 3e) and steatohepatitis, both when graded according to the non-alcoholic fatty liver disease activity score (NAS)^39^ (APRI + ALBI^high^ 13.5% vs APRI + ALBI^low^ 3.4%; *P* = 0.041; Fig. 3f) and according to Brunt et al.^40^ (APRI + ALBI^high^ 12.5% vs APRI + ALBI^low^ 1.7%; *P* = 0.020; Fig. 3g). Furthermore, a tendency toward a higher prevalence of severe SOS was found for patients above the proposed cutoff (APRI + ALBI^high^ 7.3% vs APRI + ALBI^low^ 3.4%; *P* = 0.325; Fig. 3h).

### Levels of APRI + ALBI Dynamically Change During Neoadjuvant Chemotherapy and Allow Quantification of Liver Function Recovery

**Fig. 4:**
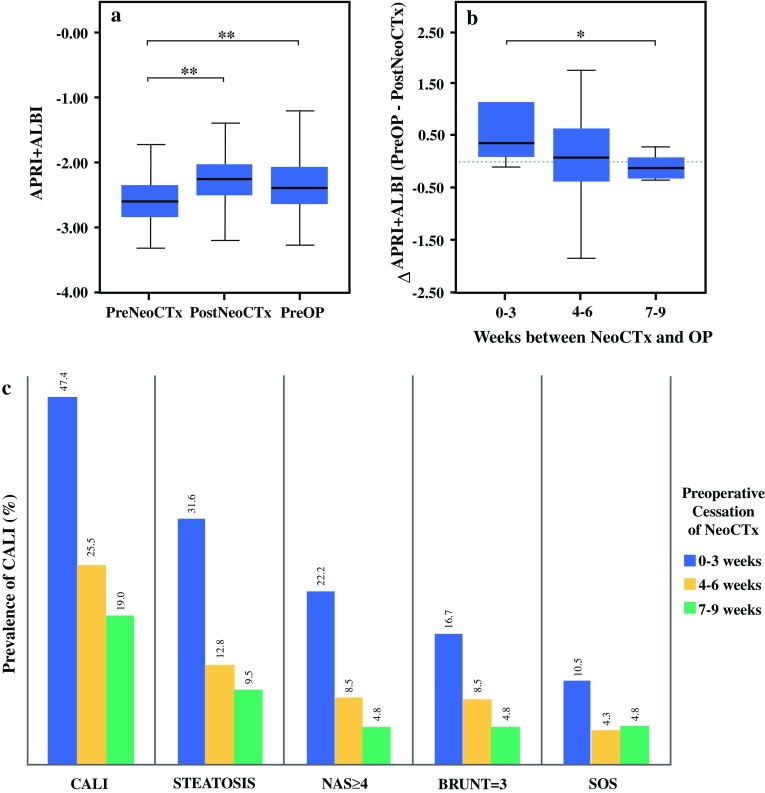
APRI + ALBI is associated with chemotherapy-associated liver injury (CALI) in patients undergoing neoadjuvant chemotherapy (NeoCTx) before liver resection. (**a**) Time course of APRI + ALBI from before NeoCTx (preNeoCTx) to completion of NeoCTx (postNeoCTx) and the preoperative time point (preOP) are shown. (**b**) Furthermore, change of APRI + ALBI from postNeoCTx to preOP (ΔAPRI + ALBI) is illustrated for patients grouped according to the length of the chemotherapy-free interval before surgery. (**c**) For the same patient groups, prevalence of CALI, steatosis, chemotherapy-associated steatohepatitis according to nonalcoholic fatty liver disease activity score (NAS) and Brunt et al. 40, and sinusoidal obstruction syndrome (SOS) is shown. *P < 0.05. **P < 0.005. APRI, aspartate aminotransferase (AST)-to-platelet ratio index; ALBI, albumin-bilirubin grade

To investigate a potential dynamic change of APRI + ALBI during NeoCTx, the score was assessed before NeoCTx and directly after completion of the last cycle. Indeed, levels of APRI + ALBI were found to increase significantly after NeoCTx (median preNeoCTx, − 2.60; median postNeoCTx, − 2.27; *P* < 0.001; Fig. 4a). This increase persisted until the time of surgery (median preNeoCTx, − 2.60; median preOP, − 2.40; *P* < 0.001; Fig. 4a). Importantly, a continuous decrease in APRI + ALBI with time between the last cycle of NeoCTx and surgery could be observed. In particular, ΔAPRI + ALBI ([APRI + ALBI{preOP}] − [APRI + ALBI {postNeoCTx)}), representing the recovery of APRI + ALBI, significantly correlated with the length of NeoCTx cessation before surgery (*P* = 0.042, *R* = − 0.305). Indeed, the decrease in APRI + ALBI was more pronounced for the patients with a period of 6 to 9 weeks after NeoCTx than for the patients who had a break shorter than 3 weeks before LR (median 0–3 weeks break, 0.35; median 7–9 weeks break, − 0.11; *P* = 0.006; Fig. 4b). Notably, this decrease in APRI + ALBI over time was paralleled by a decreased prevalence of CALI among the patients with a longer cessation of NeoCTx before surgery (Fig. 4c).

### APRI + ALBI is a Reliable Marker for Postoperative Outcome as Validated in an Independent Multicenter Cohort

To confirm the obtained data regarding the predictive potential of APRI + ALBI for postoperative outcome, a multi-center validation cohort was investigated. This analysis included 161 patients (Medical University of Vienna [*n* = 32 patients], Rudolfstiftung Hospital Vienna [*n* = 50 patients], State Hospital Wiener Neustadt [*n* = 48 patients], and Inselspital and University Hospital Bern [*n* = 31 patients]; Table S3).

**Fig. 5:**
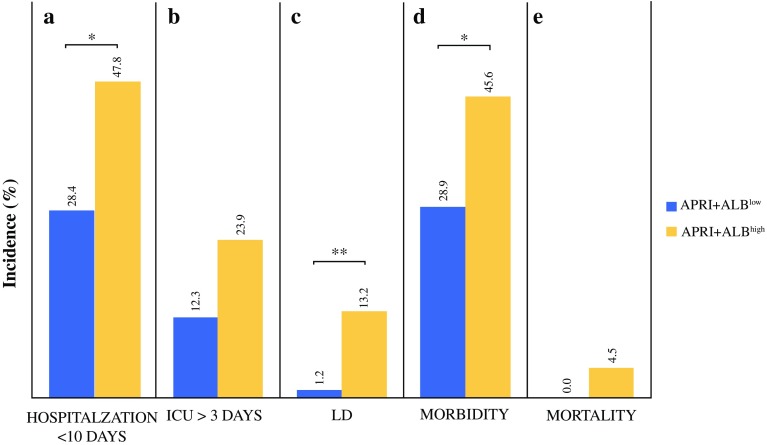
Multi-institutional validation of the predictive potential of APRI + ALBI for poor postoperative outcome. Frequencies of adverse clinical outcome are given in accordance with the proposed cutoff of –2.46 for APRI + ALBI. Incidences of (**a**) prolonged hospitalization, (**b**) prolonged intensive care unit (ICU) stay, (**c**) liver dysfunction (LD), (**d**) morbidity, and (**e**) mortality, are shown according to the risk groups (APRI + ALBI^low/high^). *P < 0.05. **P < 0.005

The patients were divided into risk groups according to their preoperative APRI + ALBI. The patients above the cutoff of –2.46 for APRI + ALBI showed a significantly longer postoperative hospital stay (APRI + ALBI^high^ 47.8% vs APRI + ALBI^low^ 28.4%; *P* = 0.015; Fig. 5a) and a tendency toward a prolonged ICU stay (APRI + ALBI^high^ 23.9% vs APRI + ALBI^low^ 12.3%; *P* = 0.066; Fig. 5b). Strikingly, the patients in the high-risk group were found to have significantly higher incidences of postoperative LD (APRI + ALBI^high^ 13.2% vs APRI + ALBI^low^ 1.2%; *P* = 0.003; Fig. 5c) and morbidity (APRI + ALBI^high^ 45.6% vs APRI + ALBI^low^ 28.9%; *P* = 0.034; Fig. 5d). Ultimately, only patients in the high-risk group died within 90 postoperative days (APRI + ALBI^high^ 4.5% vs APRI + ALBI^low^ 0.0%; *P* = 0.071; Fig. 5e).

## Discussion

For a large fraction of patients with CRCLM, NeoCTx is routinely used.^3^^,^^6^^,^^7^ Indeed, its beneficial effects have been shown for both resectable and primarily unresectable CRCLM.^3^^,^^7^^,^^41^ However, the development of CALI and a concomitantly increased risk for postoperative complications and LD remain drawbacks for NeoCTx.^16^ More importantly, reliable diagnosis and staging of CALI remain a challenging task for clinicians. Indeed, radiologic studies do not provide satisfactory information.^42^ Furthermore, proposed methods for identifying patients with CALI, such as ICG clearance,^43^^,^^44^ are expensive and have not found their way into clinical routine. Thus, liver biopsy often is the examination of choice, but due to the high degree of invasiveness, it is reserved for high-risk patients with suspicion of severe liver damage. This leaves the majority of patients undergoing LR after NeoCTx without adequate preoperative assessment for CALI.

Intriguingly, liver biopsy and histopathologic examination have been shown to have low sensitivity for most types of CALI.^45^ In a prospective study, Viganò et al.^45^ aimed to assess the sensitivity and accuracy of preoperative liver biopsies versus examination of resected liver parenchyma for CALI detection. Notably, only steatosis could be reliably detected, with a sensitivity of 88.9% and an accuracy of 93%. Importantly, diagnosis of SOS had an accuracy of only 63% and a sensitivity of only 21.1%. Similarly, steatohepatitis was diagnosed correctly in only 78% of cases and with a sensitivity of only 21.1%. This suggests that percutaneous liver biopsy does not suitably reflect the actual pathophysiologic degree of liver damage.

Strikingly, using the combination of APRI + ALBI, we were able to identify CALI (as evaluated in the surgical specimen) with a much higher sensitivity. Whereas severe steatosis was detected with a sensitivity of 90%, the cutoff was able to identify SOS with a sensitivity of 77.8%, and detection of CASH showed sensitivities of 86.7% (NAS ≥ 4) and 92.3% (Brunt = 3). This favors the use of APRI + ALBI as a noninvasive tool for the sensitive identification of CALI before surgery.

Interestingly, the patients in the high-risk group had a significantly higher prevalence of severe steatosis and CASH and a tendency toward an increased prevalence of SOS. The was paralleled by a substantially higher incidence of adverse clinical outcomes. Importantly, the gathered data could be confirmed in an independent multi-center cohort of CRCLM patients undergoing LR at four institutions. Accordingly, this leads to the suggestion that APRI + ALBI is able to stratify patients as those with clinically relevant forms of CALI and those with only slightly injured livers after NeoCTx.

Most importantly, the current study introduced APRI + ALBI as a useful tool for timing LR after NeoCTx. We present solid data on the increase in APRI + ALBI during NeoCTx, suggesting a direct correlation between the marker and induced liver damage. Interestingly, this increase was not affected by the number of NeoCTx cycles administered (*R* = − 0.115; *P* = 0.474), suggesting that the susceptibility of patients to the development of CALI might be more relevant than the duration of NeoCTx itself.

Furthermore, a decrease in APRI + ALBI during the time of chemotherapy cessation before surgery was observed. Indeed, this finding is consistent with recent reports on the amelioration of CALI after chemotherapy cessation.^46^ Correspondingly, APRI + ALBI might be a relevant tool for timing and postponement of LR. Indeed, sequential assessment of APRI + ALBI might allow dynamic monitoring of CALI and hence potentially delay surgery until the liver has adequately recovered. Although high-risk patients according to APRI + ALBI were shown to have severely reduced postoperative outcome, a transition to the low-risk group after a certain period might prevent the patient from these complications. Hence, evaluation of APRI + ALBI could be used to guide the physician’s decision concerning when to operate on a patient. However, the exact time frame might differ between patients, which makes fine-meshed monitoring of this marker essential for high-risk patients.

In conclusion, evaluation of APRI + ALBI represents an easy instrument for preoperative risk stratification of patients undergoing LR after NeoCTx. Because this marker is based on routine laboratory parameters, it is easily assessable and can readily be implicated in clinical routine. Notably, the summative combination of APRI and ALBI was found to improve the predictive potential compared with both scores assessed individually. Furthermore, evaluation of APRI + ALBI seems to allow personalized scheduling of LR after completion of NeoCTx. Ultimately, this might help to reduce the incidence of chemotherapy-associated complications after LR.

## Electronic supplementary material

Below is the link to the electronic supplementary material.

Supplementary material 1 (DOCX 552 kb)
