# Supplementary material for: The Combination of APRI and ALBI Facilitates Preoperative Risk Stratification for Patients Undergoing Liver Surgery After Neoadjuvant Chemotherapy
Source: Ann Surg Oncol. 2019 Jan 7;26(3):791–9. doi: 10.1245/s10434-018-07125-6 (PMC6373283; doi:10.1245/s10434-018-07125-6)
Supplement: Supplementary file 1 — Supplementary material 1 (DOCX 552 kb) [file 10434_2018_7125_MOESM1_ESM.docx]

**Combination of APRI and ALBI Facilitates Preoperative Risk Stratification in Patients Undergoing Liver Surgery Following Neoadjuvant Chemotherapy**

Original Article

D Pereyra^1^, B Rumpf^1^, M Ammann^2^, S Perodin^3^, D Tamandl^4^, C Haselmann^1^, J Stift^5^, C Brostjan^1^, F Laengle^2^, G Beldi^3^, T Gruenberger^6^, P Starlinger^1†^

^1^ Department of Surgery, Medical University of Vienna, General Hospital, Vienna, Austria

^2^ Department of Surgery, State Hospital Wiener Neustadt, Wiener Neustadt, Austria

^3^ Department of Visceral Surgery and Medicine, University of Bern, Inselspital, Bern, Switzerland

^4^ Department of Biomedical Imaging and Image-Guided Therapy, Medical University of Vienna, General Hospital, Vienna, Austria

^5^ Clinical Institute of Pathology, Medical University of Vienna, General Hospital, Vienna, Austria

^6^ Department of Surgery, Rudolfstiftung Hospital, Vienna, Austria – current affiliation: Department of Surgery, Kaiser Franz Josef Hospital, Vienna, Austria

† Corresponding Author:

*Patrick Starlinger, MD, PhD*

*Department of Surgery, Medical University of Vienna*

*Waehringer Guertel 18-20, 1090 Vienna, Austria*

*Tel: +43-1-40400-73526*

*E-mail:* [*patrick.starlinger@meduniwien.ac.at*](mailto:patrick.starlinger@meduniwien.ac.at)

Running Head: APRI+ALBI for monitoring of CALI

The authors declare that they do not have anything to disclose regarding conflict of interest with respect to this manuscript. No funding or financial support was received for this work.

**SUPPLEMENTAL METHODS**

***Quantification of Blood Parameters and Calculation of APRI and ALBI***

Analysis of blood parameters was performed by the clinical routine laboratory. Both, APRI and ALBI were calculated in accordance to the previously published formulas that are specified in Supplemental Fig.1. Briefly, the ratio of AST, normalized to the upper limit of normal (50 U/L at our institute), to platelet count times 100 yields the value of APRI, while ALBI is calculated by the formula −0.085×(albumin [g/L])+0.66×log(bilirubin [µmol/L]).

***Evaluation of Liver Histopathology***

In a subset of 170 patients liver histology was assessed by an experienced pathologist. Importantly, as one focus of this study was evaluation and non-invasive grading of CALI, severe steatosis (i.e. grade 3), high-grade SOS and steatohepatitis were discriminated. Steatohepatitis was diagnosed based on the non-alcoholic fatty liver disease activity score (NAS ≥ 4), as well as on the staging described by Brunt et al. (Brunt = 3)^1,2^.

***Definition and Classification of Outcome Parameters***

After surgery, patients were followed for up to 90 days. Postoperative hospitalization and stay at the intensive care unit (ICU) was assessed. Hospitalization of more than 10 days after surgery was classified as “prolonged hospitalization”, and a stay of more than 3 days at the ICU was referred to as “prolonged ICU”. Morbidity during postoperative hospital stay was classified according to Dindo et al.^3^ For classification of postoperative LD, the definition by the ISGLS was used^4^. Accordingly, patients that display elevated SB and reduced PT on the fifth postoperative day, were considered as “LD”. In addition, patients that returned to normal levels of SB and PT before the fifth day after surgery, and hence had no further blood examination, were considered as “no LD”.

***Supplemental Statistical Methods***

Differences between two groups were assessed using Mann-Whitney-U-test. For comparison of variables between two time points Wilcoxon signed-rank test was applied. Correlations between two variables were assessed using Spearmen’s Rho test. Receiver operating characteristic (ROC) analysis was conducted to assess and compare the predictive potential of APRI, ALBI and their combination for postoperative LD. Based on this ROC curve, an optimal cut-off was determined for the best variable using Youden’s J statistic. Subsequently, chi-squared test was utilized to assess differences in incidences and prevalence between the defined risk groups. Ultimately, multivariable analysis based on logistic regression including all variables being significant upon univariate analysis was fit using a stepwise forward selection model. Of note, boxplots are given without outliers to improve resolution of interquartile ranges.

**SUPPLEMENTAL FIGURES**

***Supplemental Fig.1*** Formula for calculation of APRI and ALBI.

***Supplemental Fig.2*** Levels of ALBI/APRI, APRI/ALB, APRI-ALBI, ALBI-APRI and APRIxALBI are shown for according to the postoperative outcome. While APRI - ALBI, ALBI - APRI and APRI x ALBI did not show statistically relevant differences between patients with or without postoperative morbidity, liver dysfunction (LD) or mortality, the ratio of APRI to ALBI and vice versa showed highly significant differences (APRI/ALBI: median no morbidity=-0.12, median morbidity=-0.16, P=0.035; median no LD=-0.10, median LD=-0.19, P=0.004; median no mortality=-0.12, median mortality=-0.20, P=0.021; ALBI/APRI: median no morbidity=-8.78, median morbidity=-6.21, P=0.013; median no LD=-8.35, median LD=-5.37, P=0.008; median no mortality=-8.50, median mortality=-4.93, P=0.027). *P<0.05, **P<0.005

***Supplemental Fig.3*** Incidence of postoperative LD is shown according to the grades defined by the ISGLS both for patients belonging to APRI+ALBIlow and APRI+ALBIhigh (A). Similarly, the occurrence of postoperative morbidity graded following the scheme given by Dindo et al. is shown for the mentioned risk-groups (B).

***Supplemental Fig.4*** Incidences of prolonged hospitalization (a, f), prolonged intensive care unit (ICU) stay (b, g), liver dysfunction (LD) (c, h), morbidity (d, i) and mortality (e, j), are shown according to the proposed risk groups APRI+ALBI^low/high^ for both patients undergoing minor LR (a-e) and major LR (f-j). *P<0.05, **P<0.005

**SUPPLEMENTAL TABLES**

| **Supplemental Table 1. Patient Demographics**  **(Evaluation Cohort, N=339)** | | | | | | | | |
| --- | --- | --- | --- | --- | --- | --- | --- | --- |
| **Parameter** | | | | **Median / N (% / range)** | | | | |
| **Gender** | | | |  | | | | |
| Male | | | | 214(63.1%) | | | | |
| Female | | | | 125(36.9%) | | | | |
| **Hepatic resection** | | | |  | | | | |
| Minor | | | | 175(51.6%) | | | | |
| Major | | | | 164(48.4%) | | | | |
| **Neoadjuvant Chemotherapy** | | | |  | | | | |
| No NeoCTx | | | | 42(12.4%) | | | | |
| Less Hepatotoxic | | | | 13(3.9%) | | | | |
| Capecitabine monotherapy | | | | 4(1.2%) | | | | |
| Fluorouracil + Levoleucovorin | | | | 4(1.2%) | | | | |
| Capecitabine + mitomycinC | | | | 3(0.9%) | | | | |
| Capecitabine + Bevacizumab | | | | 1(0.3%) | | | | |
| Bevacizumab monotherapy | | | | 1(0.3%) | | | | |
| Irinotecan based | | | | 51(15.0%) | | | | |
| Oxaliplatin based | | | | 222(65.5%) | | | | |
| Irinotecan+Oxaliplatin based | | | | 11(3.2%) | | | | |
| **Chemotherapy Associated Liver Injury^a^** | | | | 41(12.1%) | | | | |
| Severe steatosis | | | | 22(6.5%) | | | | |
| Steatohepatitis | | | |  | | | | |
| NAS ^2^ ≥ 4 | | | | 16(4.7%) | | | | |
| Brunt ^1^ = 3 | | | | 14(4.1%) | | | | |
| Severe sinusoidal dilatation | | | | 11(3.2%) | | | | |
| **Preoperative Parameters** | | | |  | | | | |
| PDR(%) | | | | 20.0(3.5-36.4) | | | | |
| R15(%) | | | | 6.0(1.0-31.0) | | | | |
| Platelets(x10^3^/µl) | | | | 157(49-503) | | | | |
| SB(mg/dl) | | | | 0.64(0.19-2.87) | | | | |
| PT(%) | | | | 106(43-150) | | | | |
| AP(U/l) | | | | 105(40-1111) | | | | |
| GGT(U/l) | | | | 45(11-968) | | | | |
| AST(U/l) | | | | 29(5-1114) | | | | |
| ALT(U/l) | | | | 23(2-605) | | | | |
| Albumin(g/l) | | | | 41.2(22.2-53.7) | | | | |
| Age(years) | | | | 63(28-88) | | | | |
| **Postoperative Outcome** | | | |  | | | | |
| Postoperative hospitalization (days) | | | | 8(4-77) | | | | |
| Postoperative ICU stay (days) | | | | 1(0-24) | | | | |
| ISGLS liver dysfunction^4^ | | | | 37(10.9%) | | | | |
| ISGLS A | | | | 24(7.1%) | | | | |
| ISGLS B | | | | 7(2.1%) | | | | |
| ISGLS C | | | | 6(1.8%) | | | | |
| Morbidity (Dindo et al.^3^) | | | | 123(36.3%) | | | | |
| Mortality (within 90 days) | | | | 7(2.1%) | | | | |
| NeoCTx=neoadjuvant chemotherapy, PDR=plasma disappearance rate, R15=retention rate at 15 minutes, SB=serum bilirubin, PT=prothrombin time, AP=alkaline phosphatase, GGT=gamma-glutamyl transferase, AST=aspartate aminotransferase, ALT=alanine aminotransferase, ICU=intensive care unit, NA=not assessed.  ^a^ Histology was assessed in a subgroup of 170 patients. | | | | | | | | |
| **Supplemental Table 2. Multivariable Analysis for Liver Dysfunction** | | | | | | | | |
|  | **Univariate Analysis** | | | | **Multivariable Analysis** | | | |
| **Parameter** | **OR** | **95%-CI** | **P-Value** | | | **OR** | **95%-CI** | **P-Value** |
| **APRI+ALBI > -2.46** | **6.685** | **2.290**-**19.512** | **0.001** | | | **5.501** | **1.822-16.606** | **0.002** |
| Gender | 0.917 | 0.449-1.875 | 0.813 | | |  |  |  |
| **Extent of hepatic resection** | **2.833** | **1.351-5.942** | **0.006** | | |  |  |  |
| NeoCTx | 2.625 | 0.607-11.350 | 0.196 | | |  |  |  |
| Type of NeoCTx | 1.521 | 0.982-2.354 | 0.060 | | |  |  |  |
| Preoperative Parameters |  |  |  | | |  |  |  |
| PDR(%) | 0.943 | 0.875-1.016 | 0.124 | | |  |  |  |
| **R15(%)** | **1.103** | **1.015-1.198** | **0.021** | | |  |  |  |
| Platelets(x10^3^/µl) | 0.995 | 0.989-1.000 | 0.070 | | |  |  |  |
| SB(mg/dl) | 1.043 | 0.449-2.422 | 0.922 | | |  |  |  |
| PT(%) | 0.991 | 0.974-1.008 | 0.295 | | |  |  |  |
| **AP(U/l)** | **1.003** | **1.000-1.006** | **0.047** | | |  |  |  |
| **GGT(U/l)** | **1.005** | **1.002-1.008** | **0.002** | | | **1.006** | **1.003-1.010** | **<0.001** |
| AST(U/l) | 0.999 | 0.993-1.005 | 0.764 | | |  |  |  |
| ALT(U/l) | 0.999 | 0.992-1.006 | 0.822 | | |  |  |  |
| Albumin(g/l) | 0.932 | 0.864-1.005 | 0.068 | | |  |  |  |
| Age(years) | 1.023 | 0.988-1.060 | 0.192 | | |  |  |  |
| NeoCTx=neoadjuvant chemotherapy, PDR=plasma disappearance rate, R15=retention rate at 15 minutes, SB=serum bilirubin, PT=prothrombin time, AP=alkaline phosphatase, GGT=gamma-glutamyl transpeptidase, AST=aspartate aminotransferase, ALT=alanine aminotransferase. | | | | | | | | |

| **Supplemental Table 3. Patient Demographics**  **(Validation Cohort, N=161)** | |  |
| --- | --- | --- |
| **Parameter** | **Median / N (% / range)** | |
| **Gender** |  | |
| Male | 113(70.2%) | |
| Female | 48(29.8%) | |
| **Hepatic resection** |  | |
| Minor | 100(62.1%) | |
| Major | 61(37.9%) | |
| **Preoperative Parameters** |  | |
| PDR(%) | 18.0(13.0-30.0) | |
| R15(%) | 6.0(1.0-16.0) | |
| Platelets(x10^3^/µl) | 214(77-487) | |
| SB(mg/dl) | 0.54(0.11-2.30) | |
| PT(%) | 100(37-150) | |
| AP(U/l) | 88(32-296) | |
| GGT(U/l) | 47(7-562) | |
| AST(U/l) | 26(6-89) | |
| ALT(U/l) | 23(4-117) | |
| Albumin(g/l) | 40.9(25.5-59.0) | |
| Age(years) | 64(29-88) | |
| **Postoperative Outcome** |  | |
| Postoperative hospitalization (days) | 9(3-59) | |
| Postoperative ICU stay (days) | 1(0-19) | |
| ISGLS liver dysfunction^4^ | 10(6.2%) | |
| ISGLS A | 3(1.9%) | |
| ISGLS B | 3(1.9%) | |
| ISGLS C | 4(2.5%) | |
| Morbidity (Dindo et al.^3^) | 56(34.8%) | |
| Mortality (within 90 days) | 3(1.9%) | |
| PDR=plasma disappearance rate, R15=retention rate at 15 minutes, SB=serum bilirubin, PT=prothrombin time, AP=alkaline phosphatase, GGT=gamma-glutamyl transferase, AST=aspartate aminotransferase, ALT=alanine aminotransferase, ICU=intensive care unit. | |  |

**REFERENCES**

**1.** Brunt EM, Janney CG, Di Bisceglie AM, Neuschwander-Tetri BA, Bacon BR. Nonalcoholic steatohepatitis: a proposal for grading and staging the histological lesions. *The American journal of gastroenterology.* Sep 1999;94(9):2467-2474.

**2.** Kleiner DE, Brunt EM, Van Natta M, et al. Design and validation of a histological scoring system for nonalcoholic fatty liver disease. *Hepatology (Baltimore, Md.).* Jun 2005;41(6):1313-1321.

**3.** Dindo D, Demartines N, Clavien PA. Classification of surgical complications: a new proposal with evaluation in a cohort of 6336 patients and results of a survey. *Annals of surgery.* Aug 2004;240(2):205-213.

**4.** Rahbari NN, Garden OJ, Padbury R, et al. Posthepatectomy liver failure: a definition and grading by the International Study Group of Liver Surgery (ISGLS). *Surgery.* May 2011;149(5):713-724.
